# *BCL3-PVRL*2*-TOMM40* SNPs, gene-gene and gene-environment interactions on dyslipidemia

**DOI:** 10.1038/s41598-018-24432-w

**Published:** 2018-04-18

**Authors:** Liu Miao, Rui-Xing Yin, Shang-Ling Pan, Shuo Yang, De-Zhai Yang, Wei-Xiong Lin

**Affiliations:** 1 Department of Cardiology, Institute of Cardiovascular Diseases, The First Affiliated Hospital, Guangxi Medical University, Nanning, 530021 Guangxi People’s Republic of China; 2 Department of Pathophysiology, School of Premedical Science, Guangxi Medical University, Nanning, 530021 Guangxi People’s Republic of China; 3 Department of Molecular Genetics, Medical Scientific Research Center, Guangxi Medical University, Nanning, 530021 Guangxi People’s Republic of China

## Abstract

Little is known about the association of the *BCL3-PVRL2-TOMM40* SNPs and dyslipidemia. This study was to detect 12 *BCL3-PVRL2-TOMM40* SNPs, gene-gene and gene-environment interactions on dyslipidemia in the Chinese Maonan population. Genotyping was performed in 1130 normal and 832 dyslipidemia participants. Generalized multifactor dimensionality reduction was used to screen the best interaction combination among SNPs and environmental exposures. Allele and genotype frequencies of the detected SNPs were different between the two groups (*P* < 0.05–0.001). Association of the 12 SNPs and serum lipid levels was observed (*P* < 0.004–0.001). Multiple-locus linkage disequilibrium was not statistically independent in the population (*D’* = 0.01–0.98). The dominant model of rs8100239 and rs157580 SNPs, several haplotypes and G × G interaction haplotypes contributed to a protection, whereas the dominant model of rs10402271, rs3810143, rs519113, rs6859 SNPs, another haplotypes and G × G interaction haplotypes revealed an increased morbidity function (*P* < 0.05–0.001). There were significant three-locus model involving SNP-SNP, SNP-environment, haplotype-haplotype interactions (*P* < 0.05–0.001). The subjects carrying several genotypes and haplotypes decreased dyslipidemia risk, whereas the subjects carrying other genotypes and haplotypes increased dyslipidemia risk. The *BCL3-PVRL2-TOMM40* SNPs, gene-gene and gene-environment interactions on dyslipidemia were observed in the Chinese Maonan population.

## Introduction

Atherosclerotic cardiovascular disease (ASCVD) and its clinical manifestations, such as myocardial infarction (MI) and ischemic stroke, are the leading cause of morbidity and mortality throughout the world^1^. Multiple exposures just as genetic and environmental factors have been associated with an increased risk of cardiovascular events^2^, such as sex, age, dyslipidemia, hypertension, diabetes, smoking behavior, and family history^3–5^. As we know that the main point for ASCVD pathophysiologic mechanisms is atherosclerosis^6^, and dyslipidemia is the leading cause of atherosclerosis^7^. Recently, the compelling genes for modifying lipid metabolism emerged from very large replicated genome-wide association studies (GWASes): the B-cell CLL/lymphoma 3 gene (*BCL3* [MIM109560]), the poliovirus receptor-related 2 gene (*PVRL2* [MIM600798]) and the translocase of outer mitochondrial membrane gene (*TOMM40* [MIM608061]), those of them can give rise to dyslipidemia^8–11^.

China is a multi-ethnic country, including 56 nationalities. Han is the largest one and Maonan is one of the 55 minorities with a population of 107,166 (Rank 37) according to the sixth national census statistics of China in 2010. Recent phylogenetic and principal component analyses revealed that the Maonan people belong to the Southeastern Asian group and are most closely related to the Buyi people^12^ and the genetic relationship between Maonan nationality and other minorities in Guangxi^13^ was much closer than that between Maonan and Han nationalities^14^. In a previous study, we have found that the *BRCA2* rs9534275 SNP modulated serum total cholesterol (TC), low-density lipoprotein cholesterol (LDL-C), apolipoprotein (Apo) B levels, and the ApoA1/ApoB ratio in the hypercholesterolemic subjects^15^, but little is known about the relationship between dyslipidemia and other gene polymorphisms. Therefore, the objective of this study was to detect the association of 12 *BCL3-PVRL2-TOMM40* SNPs, their haplotypes and G × G interactions with serum lipid phenotypic variations in the Maonan population. In addition, we wanted to use multifactor dimensionality reduction (MDR) to test the association analysis of these loci based on haplotype clusters, G × G and G × E interactions on dyslipidemia in this population.

## Results

### Demographic and biochemical characteristics

The demographic, epidemiological and clinical characteristics in 1,962 study subjects are summarized in Table 1. The levels of weight, percent of smoking and drinking, serum glucose, serum TC, triglyceride (TG) and LDL-C were higher, as well as the levels of serum high-density lipoprotein cholesterol (HDL-C) and the ratio of ApoA1 to ApoB were lower in dyslipidemia than in normal groups (*P* < 0.05–0.001). However, no difference was observed in age, sex, height, body mass index (BMI), waist circumference (WC), systolic blood pressure (SBP), diastolic blood pressure (DBP), pulse pressure (PP), serum ApoA1 and ApoB levels between the two groups (*P* > 0.05 for all).

**Table 1:** Comparison of demographic, lifestyle characteristics and serum lipid levels between the normal and dyslipidemia populations in Maonan minority.

Parameter	Normal	Dyslipidemia	*test-statistic*	*P*
**Number**	**1130**	**832**		
Male/female	428/702	316/516	0.002	0.962
Age (years)^a^	56.37 ± 10.78	57.22 ± 11.12	1.682	0.195
Height (cm)	153.70 ± 7.93	154.93 ± 7.89	0.672	0.412
Weight (kg)	52.18 ± 9.69	57.98 ± 10.23	3.839	0.048
Body mass index (kg/m^2^)	22.02 ± 3.44	24.03 ± 3.05	0.258	0.612
Waist circumference (cm)	74.98 ± 8.28	82.39 ± 8.61	1.796	0.180
Smoking status [*n* (%)]				
Non-smoker	877(77.61)	606(72.84)		
≤20 cigarettes/day	56(4.96)	42(5.05)		
>20 cigarettes/day	197(17.43)	184(22.11)	6.862	0.032
Alcohol consumption [*n* (%)]				
Non-drinker	918(81.24)	593(71.27)		
≤25 g/day	68(6.02)	44(5.29)		
>25 g/day	144 (12.74)	195(23.44)	38.342	4.72E-009
Systolic blood pressure (mmHg)	132.20 ± 22.82	139.78 ± 22.59	0.912	0.340
Diastolic blood pressure (mmHg)	81.60 ± 11.86	86.16 ± 12.21	2.311	0.127
Pulse pressure (mmHg)	50.60 ± 17.20	53.62 ± 18.71	2.169	0.141
Glucose (mmol/L)	6.12 ± 1.44	6.54 ± 2.20	21.784	3.00E-006
Total cholesterol (mmol/L)	4.79 ± 0.96	6.13 ± 1.09	4.174	0.041
Triglyceride (mmol/L)^b^	1.23(0.81)	3.46(1.91)	214.26	2.00E-013
HDL-C (mmol/L)	1.69 ± 0.48	1.50 ± 0.40	14.162	1.73E-004
LDL-C (mmol/L)	2.76 ± 0.77	3.48 ± 0.97	17.218	3.50E-005
ApoA1 (g/L)	1.37 ± 0.29	1.38 ± 0.26	0.814	0.367
ApoB (g/L)	0.82 ± 0.19	1.10 ± 0.20	0.178	0.673
ApoA1/ApoB	1.73 ± 0.53	1.29 ± 0.42	38.512	6.62E-010

*HDL-C*, high-density lipoprotein cholesterol; *LDL-C*, low-density lipoprotein cholesterol; *Apo*, Apolipoprotein. ^a^Mean ± SD determined by *t*-test. ^b^Because of not normally distributed, the value of triglyceride was presented as median (interquartile range), the difference between the two groups was determined by the Wilcoxon-Mann-Whitney test.

### Genotype and allele frequencies and the association with serum lipid levels

**Figure 1:**
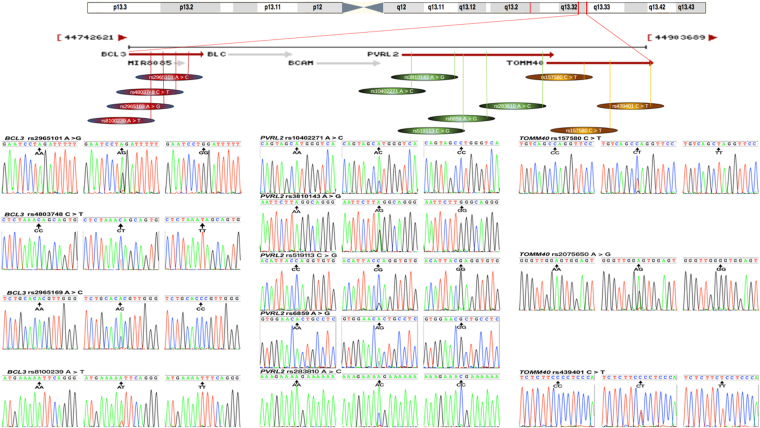
The positions and parts of the nucleotide direct sequencing results of the *BCL3*, *PRVL*2 and *TOMM40* genes SNPs. *BCL3*, the B-cell CLL/lymphoma 3 gene; *PVRL2*, the poliovirus receptor-related 2 gene; *TOMM40*, the translocase of outer mitochondrial membrane 40 gene.

**Figure 2:**
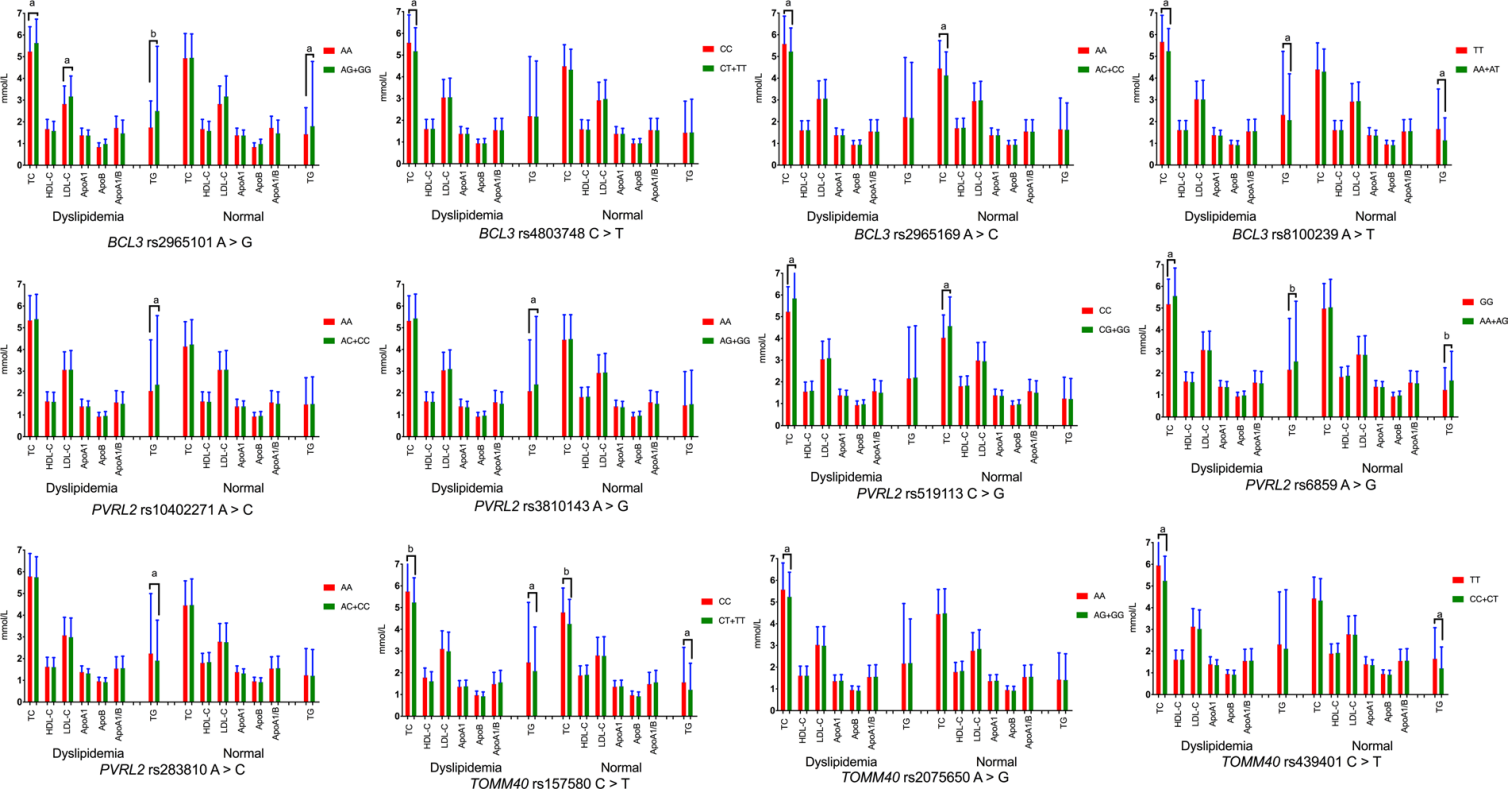
Association between the genotypes of *BCL3*, *PVRL2* and *TOMM40* SNPs and serum lipid levels in the normal and dyslipidemia individuals. *TC*, Total cholesterol; *TG*, Triglyceride; *HDL-C*, High-density lipoprotein cholesterol; *LDL-C*, Low-density lipoprotein cholesterol; *Apo*, Apolipoprotein. ^a^*P* < 0.004; ^b^*P* < 0. 001. (The *P*-value less than 0.004 was considered statistically significant after adjusting by Bonferroni correction).

The detected 12 mutations in this motif are located in a closely genomic region of chromosome 19 (Fig. 1). As shown in Table 2, the genotype and allele frequencies of these variants were different between the two groups (*P* < 0.05–0.001). All mutations exhibited the Hardy-Weinberg equilibrium (HWE, *P* > 0.05 for all). In the meantime, the dominant model of rs8100239 and rs157580 SNPs contributed to a protection, whereas the dominant model of rs10402271, rs3810143, rs519113 and rs6859 SNPs revealed an increased morbidity function (*P* < 0.05–0.001). As shown in Fig. 2, we discovered the association of the *BCL3, PVRL2* and *TOMM40* mutations with TC (rs2965101, rs4803748, rs2965169, rs8100239, rs519113, rs6859, rs157580, rs2075650 and rs439401), TG (rs2965101, rs8100239, rs10402271, rs3810143, rs6859, rs283810 and rs157580), LDL-C (rs2965101) in dyslipidemia group; and with TC (rs2965169, rs519113 and rs157580), TG (rs2965101, rs8100239, rs6859 and rs157580) in the normal group (*P* < 0.004–0.001); respectively.

**Table 2:** The association between the *BCL3, PVRL2, TOMM40* polymorphisms with dyslipidemia [n (%)].

Mutation	Genotype	Normal (n = 1130)	Dyslipidemia (n = 832)	${\boldsymbol{\chi }}$ ^2^	*P*-value	OR (95% CI)	^*^P-value
*BCL3* rs2965101 A > G	AA	488(43.19)	400(48.08)	4.627	0.035	1	—
	AG + GG	642(56.81)	432(51.92)			0.96(0.79–1.16)	0.67
	MAF	562(24.87)	506(30.41)	14.856	1.6E-004		
	*P*_*HWE*_	0.862	0.102				
*BCL3* rs4803748 C > T	CC	454(40.18)	294(35.34)	4.759	0.031	1	—
	CT + TT	676(59.82)	538(64.66)			0.91(0.75–1.07)	0.20
	MAF	836(36.99)	668(40.14)	4.031	0.045		
	*P*_*HWE*_	0.371	0.093				
*BCL3* rs2965169 A > C	AA	482(42.65)	312(37.50)	5.285	0.023	1	—
	AC + CC	648(57.35)	520(62.50)			0.88(0.72–1.11)	0.37
	MAF	714(31.59)	624(37.50)	14.822	1.1E-004		
	*P*_*HWE*_	0.112	0.066				
*BCL3* rs8100239 A > T	TT	585(51.77)	488(58.65)	9.164	0.002	1	—
	AT + AA	545(48.23)	344(41.35)			0.74(0.61–0.90)	0.002
	MAF	591(26.15)	378(22.71)	5.086	0.024		
	*P*_*HWE*_	0.051	0.233				
*PVRL2* rs10402271 A > C	AA	824(72.92)	560(67.30)	7.264	0.007	1	—
	AC + CC	306(27.07)	272(32.70)			1.25(1.01–1.54)	0.037
	MAF	330(14.60)	288(17.30)	5.288	0.021		
	*P*_*HWE*_	0.843	0.427				
*PVRL2* rs3810143 A > G	AA	825(73.00)	536(64.42)	16.624	4.6E-005	1	—
	AG + GG	305(20.00)	296(35.58)			1.43(1.17–1.76)	6E-004
	MAF	341(15.09)	320(19.23)	11.74	0.001		
	*P*_*HWE*_	0.473	0.152				
*PVRL2* rs519113 C > G	CC	745(65.93)	536(60.58)	5.934	0.015	1	—
	CG + GG	305(34.07)	296(39.42)			1.33(1.09–1.62)	0.0048
	MAF	397(17.57)	368(22.11)	12.636	3.8E-004		
	*P*_*HWE*_	0.198	0.073				
*PVRL2* rs6859 A > G	GG	563(49.82)	344(41.70)	13.852	2E-004	1	—
	AG + AA	567(50.18)	488(58.30)			1.23(1.01–1.49)	0.038
	MAF	687(30.40)	648(38.94)	31.168	2.3E-008		
	*P*_*HWE*_	0.221	0.143				
*PVRL2* rs283810 A > C	AA	884(78.23)	610(73.32)	6.367	0.012	1	—
	AC + CC	246(21.77)	222(26.68)			0.64(0.53–0.83)	0.084
	MAF	258(11.42)	246(14.78)	9.71	0.002		
	*P*_*HWE*_	0.488	0.098				
*TOMM40* rs157580 C > T	CC	201(17.78)	280(33.65)	65.185	1E-013	1	—
	CT + TT	929(82.22)	552(66.35)			0.92(0.88–1.36)	0.026
	MAF	859(38.00)	749(45.01)	19.434	1E-005		
	*P*_*HWE*_	0.051	0.893				
*TOMM40* rs2075650 A > G	AA	646(57.16)	426(51.20)	6.882	0.009	1	—
	AG + GG	484(42.84)	406(48.80)			1.03(0.78–1.23)	0.87
	MAF	550(24.34)	473(28.42)	8.315	0.004		
	*P*_*HWE*_	0.899	0.564				
*TOMM40* rs439401 C > T	TT	314(27.79)	344(41.34)	39.521	3.3E-009	1	—
	CT + CC	816(72.21)	488(58.66)			0.78(0.56–1.02)	0.061
	MAF	901(39.86)	744(44.71)	9.237	0.002		
	*P*_*HWE*_	0.089	0.234				

*BCL3*, the B-cell CLL/lymphoma 3 gene; *PVRL2*, the poliovirus receptor-related 2 gene; *TOMM40*, the translocase of outer mitochondrial membrane 40 gene; *HWE*, Hardy-Weinberg equilibrium. *MAF*, minor allele frequency. *P*-value defined as Chi-square test probability. ^*^P-value defined as Logistic test probability.

### Haplotype-based association with dyslipidemia

**Figure 3:**
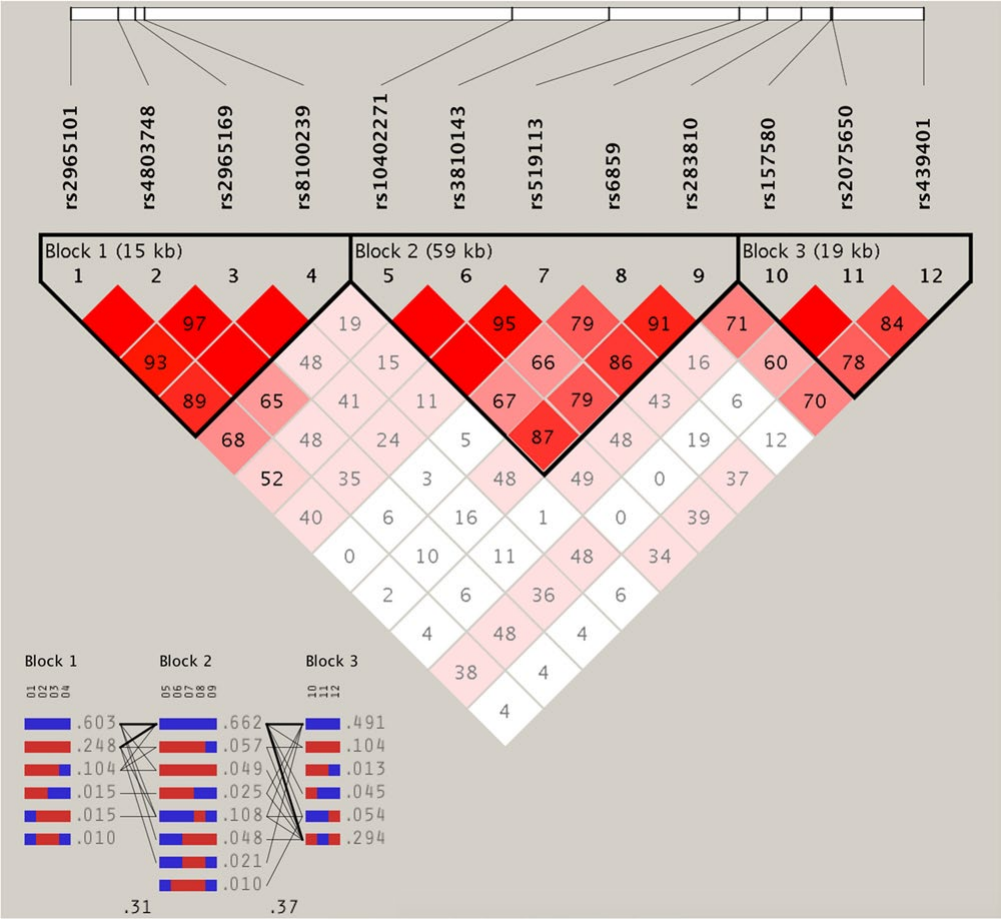
The linkage disequilibrium (LD) represents pair-wise *D’* in the combined population of normal and dyslipidemia.

Multiple-locus linkage disequilibrium (LD) elucidated that the detected sites were not statistically independent in this study population. Figure 3 showed the LD blocks and the haplotypes for blocks combined in two groups. As shown in Table 3, the commonest haplotypes were *BCL3* A-C-A-T, *PVRL2* A-A-G-G-A and *TOMM40* T-A-T (>30% of the samples). The frequencies of the *BCL3* A-T-C-A, *BCL3* A-T-C-T, *PVRL2* A-A-C-A-A, *PVRL2* A-A-C-A-C, *PVRL2* A-A-G-G-A, *PVRL2* C-G-C-A-A, *PVRL2* C-G-C-A-C, *TOMM40* C-A-C, *TOMM40* C-A-T, and *TOMM40* T-A-T haplotypes were quantitative significantly different between the dyslipidemia and normal groups. At the same time, the haplotypes of *BCL3* A-T-C-A, *PVRL2* A-A-C-A-C, *PVRL2* A-A-G-G-A and *TOMM40* T-A-T contributed to a protection, whereas the haplotypes of *BCL3* A-T-C-T, *PVRL2* A-A-C-A-A, *PVRL2* C-G-C-A-A, *PVRL2* C-G-C-A-C, *TOMM40* C-A-C and *TOMM40* C-A-T revealed an increased morbidity function (*P* < 0.05–0.001, respectively).

**Table 3:** Prevalence of haplotype frequencies in the dyslipidemia and normal populations [n (frequency)].

NO.	Haplotype	Dyslipidemia	Normal	χ^**2**^	*P*-value	Odd Ratio [95% CI]
B1	*BCL3* A-C-A-T	938.58(0.564)	1229.53(0.544)	1.102	0.293854	1.071[0.942~1.129]
B2	*BCL3* A-T-C-A	96.66(0.058)	187.88(0.083)	9.258	0.002354	0.675[0.524~0.871]
B3	*BCL3* A-T-C-T	124.42(0.075)	125.60(0.056)	5.689	0.017098	1.365[1.056~1.764]
B4	*BCL3* G-C-A-T	77.42(0.047)	133.47(0.059)	3.116	0.077544	0.772[0.579~1.030]
B5	*BCL3* G-T-C-A	311.34(0.187)	403.12(0.178)	0.376	0.539673	1.053[0.893~1.240]
B6	*BCL3* G-T-C-T	91.59(0.055)	134.41(0.059)	0.405	0.524790	0.915[0.696~1.203]
P1	*PVRL2* A-A-C-A-A	113.18(0.068)	48.53(0.021)	56.535	5.77E-014	3.471[2.463~4.892]
P2	*PVRL2* A-A-C-A-C	28.34(0.017)	107.75(0.049)	26.044	3.40E-007	0.352[0.231~0.534]
P3	*PVRL2* A-A-G-A-A	180.43(0.108)	248.12(0.110)	0.070	0.790859	1.028[0.838~1.261]
P4	*PVRL2* A-A-G-G-A	936.62(0.563)	1486.80(0.658)	24.416	7.90E-009	0.705[0.613~0.810]
P5	*PVRL2* C-G-C-A-A	127.14(0.076)	126.64(0.056)	8.292	0.003994	1.453[1.125~1.876]
P6	*PVRL2* C-G-C-A-C	125.90(0.076)	110.17(0.049)	14.533	1.39E-004	1.666[1.278~2.171]
T1	*TOMM40* C-A-C	649.30(0.390)	662.25(0.293)	41.472	1.24E-010	1.553[1.358~1.776]
T2	*TOMM40* C-A-T	245.91(0.148)	102.25(0.045)	125.224	4.92E-029	3.673[2.889~4.671]
T3	*TOMM40* T-A-C	22.72(0.014)	121.31(0.054)	1.273	0.273542	0.834[0.736~0.944]
T4	*TOMM40* T-A-T	482.07(0.310)	1111.18(0.492)	162.507	1.78E-032	0.420[0.367~0.480]

The haplotype is combined with *BCL3* rs2965101-rs4803748-rs2965169-rs8100239, *PVRL2* rs10402271-rs3810143-rs519113-rs6859-rs283810 and *TOMM40* rs157580-rs2070650-rs439401. *BCL3*, the B-cell CLL/lymphoma 3 gene; *PVRL2*, the poliovirus receptor-related 2 gene; *TOMM40*, the translocase of outer mitochondrial membrane 40 gene; Rare Hap (frequency < 1%) in both populations has been dropped.

### G × G interaction-based association with dyslipidemia

As shown in Table 4, the commonest G × G interaction was A-C-A-T-A-A-G-G-A-T-A-T (>15% of the samples). The frequencies of the A-C-A-T-A-A-G-A-A-T-A-T, A-C-A-T-A-A-G-G-A-C-A-C, A-C-A-T-A-A-G-G-A-C-A-T, A-C-A-T-A-A-G-G-A-T-A-T, A-T-C-A-A-A-G-G-A-T-A-T, G-T-C-A-A-A-G-A-A-C-A-C, G-T-C-A-A-A-G-G-A-C-A-C and G-T-C-A-A-A-G-G-A-T-A-T G × G interactions were significantly different between the two groups. In the meantime, the G × G interaction haplotypes of A-C-A-T-A-A-G-A-A-T-A-T, A-T-C-A-A-A-G-G-A-T-A-T, and G-T-C-A-A-A-G-G-A-T-A-T resulted in a protection, whereas the G × G interaction haplotypes of A-C-A-T-A-A-G-G-A-C-A-C, A-C-A-T-A-A-G-G-A-C-A-T, A-C-A-T-A-A-G-G-A-T-A-T, G-T-C-A-A-A-G-A-A-C-A-C and G-T-C-A-A-A-G-G-A-C-A-C revealed an increased morbidity function (*P* < 0.01–0.001).

**Table 4:** Prevalence of G × G interaction frequencies in the dyslipidemia and normal populations [n (frequency)].

No.	G × G inteactions												Dyslipidemia	Normal	**χ** ^**2**^	*P*-value	Odd Ratio [95%CI]
	A	B	C	D	E	F	G	H	I	J	K	L					
H1	A	C	A	T	A	A	G	A	A	T	A	T	16.00(0.010)	114.53(0.051)	36.149	1.96E-009	0.224[0.132~0.380]
H2	A	C	A	T	A	A	G	G	A	C	A	C	275.21(0.165)	151.33(0.067)	174.17	1.89E-015	4.125[3.372~5.268]
H3	A	C	A	T	A	A	G	G	A	C	A	T	94.23(0.057)	56.53(0.025)	45.707	1.53E-015	3.100[2.202~4.365]
H4	A	C	A	T	A	A	G	G	A	T	A	T	179.69(0.158)	501.71(0.22)	46.994	7.99E-012	1.502[1.411~1.612]
H5	A	T	C	A	A	A	G	G	A	T	A	T	18.38(0.011)	67.83(0.030)	9.311	0.002266	0.451[0.268~0.761]
H6	G	T	C	A	A	A	G	A	A	C	A	C	53.22(0.032)	12.63(0.006)	56.340	7.09E-014	7.656[4.119~14.232]
H7	G	T	C	A	A	A	G	G	A	C	A	C	51.08(0.031)	52.33(0.023)	7.313	0.006869	1.717[1.156~2.550]
H8	G	T	C	A	A	A	G	G	A	T	A	T	49.41(0.030)	149.04(0.08)6)	32.3688	1.35E-008	0.399[0.288~0.552]

A, *BCL3* rs2965101 A > G; B, *BCL3* rs4804748 C > T; C, *BCL3* rs2965169 A > C; D, *BCL3* rs8100239 A > T; E, *PVRL2* rs10402271 A > C; F, *PVRL2* rs3810143 A > G; G, *PVRL2* rs519113 C > G; H, *PVRL2* rs6859 A > G; I, *PVRL2* rs283810 A > C; J, *TOMM40* rs157580 C > T; K, *TOMM40* rs2075650 A > G; L, *TOMM40* rs439401 C > T. *BCL3*, the B-cell CLL/lymphoma 3 gene; *PVRL2*, the poliovirus receptor-related 2 gene; *TOMM40*, the translocase of outer mitochondrial membrane 40 gene. Rare Hap (frequency < 1%) in both populations has been dropped.

### Gene-gene and gene-environment interaction on dyslipidemia

**Figure 4:**
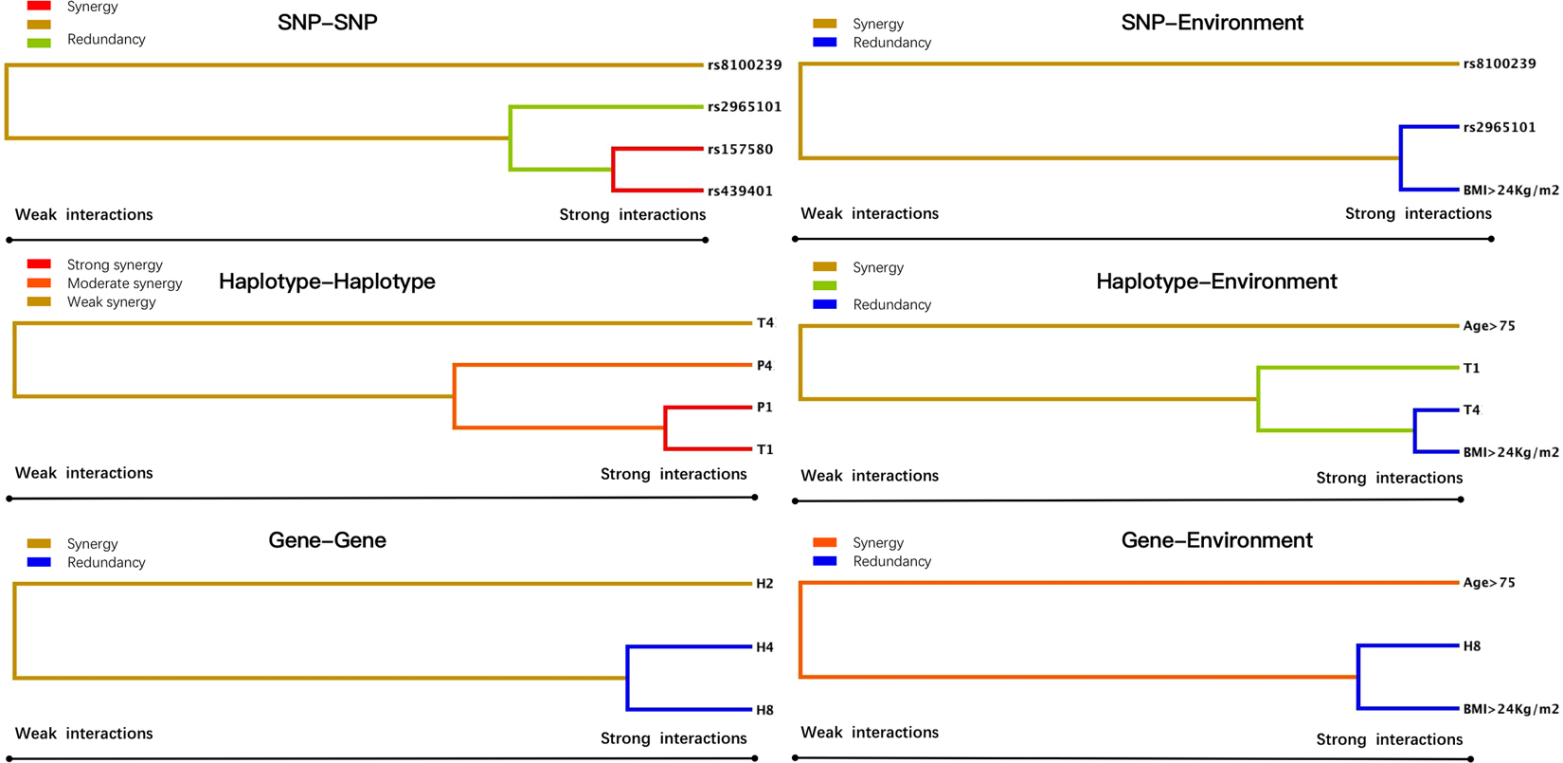
Different types of interaction dendrogram. The strongly interacting elements appear close together at the leaves of the of tree, and the weakly interacting elements appear distant from each other.

GMDR model was used to assess the impact of the gene-gene and gene-environment exposures including age, sex, BMI, blood pressure, serum glucose, smoking and drinking interaction on dyslipidemia risk, after adjustment for covariates. Table 5 summarized the results obtained from GMDR analysis for two- to three-locus models for gene-gene interaction and two- to three-locus models for gene-environment interaction. There was a significant three-locus model (*P* < 0.001) involving rs2965101, rs157580 and rs439401 SNPs, indicating a potential SNP-SNP interaction among rs296510, rs157580 and rs439401 SNPs. Overall, this model had a cross-validation consistency of 10 of 10, and had the testing accuracy of 74.94%. A significant three-locus model (*P* < 0.001) involving rs2965101, rs8100239 SNPs and BMI > 24 kg/m^2^ was also found, indicating a potential SNP-environment interaction and this model had a cross-validation consistency of 10 of 10, and had the testing accuracy of 70.11%. Besides these, three-locus model had been detected about haplotype-haplotype interaction (*PVRL2* A-A-G-G-A, *TOMM40* C-A-C and *TOMM40* T-A-T) and haplotype-environment interaction (*TOMM40* C-A-C, *TOMM40* T-A-T and BMI > 24 kg/m^2^, *P* < 0.001, respectively). Similarly, in gene-gene interaction (A-C-A-T-A-A-G-G-A-C-A-C, A-C-A-T-A-A-G-G-A-T-A-T and G-T-C-A-A-A-G-G-A-T-A-T) and gene-environment interaction (G-T-C-A-A-A-G-G-A-T-A-T, age > 75 and BMI > 24 kg/m^2^). Entropy-based interaction dendrogram, built by MDR is shown in Fig. 4, showed the strongest synergy between rs157580 and rs439401 in SNP-SNP interaction and *PVRL2* A-A-C-A-A and *TOMM40* C-A-C in haplotype-haplotype interaction. However, the redundancy effect can be found in SNP-environment interaction (rs2965101 and BMI > 24 kg/m^2^), haplotype-environment interaction (*TOMM40* T-A-T and BMI > 24 kg/m^2^), gene-gene interaction (A-C-A-T-A-A-G-G-A-T-A-T and G-T-C-A-A-A-G-G-A-T-A-T) and haplotype-environment interaction (G-T-C-A-A-A-G-G-A-T-A-T and BMI > 24 kg/m^2^). In order to obtain the odds ratios (OR) and 95% confidence interval (CI) for the joint effects, we conducted interaction analysis by using logistic regression (Table 6). We found that the subjects with rs157580 CT/TT and rs439401 CC/CT genotypes have the lowest dyslipidemia risk (adjusted OR = 0.54, 95% CI = 0.32–0.93, *P* < 0.001) compared to the subjects with rs157580 CC and rs439401 TT genotype, respectively. When considered with SNP-environment interaction, we found that the subjects with rs2965101AC/CC genotypes and BMI > 24 kg/m^2^ increased dyslipidemia risk (adjusted OR = 1.08, 95% CI = 0.84–1.44, *P* = 0.0015). However, when haplotype-haplotype and haplotype-environment interactions were analyzed, we could find that the *PVRL2* A-A-C-A-A and *TOMM40* C-A-C carriers (adjusted OR = 5.47, 95% CI = 3.64–7.73, *P* < 0.001) and *TOMM40* T-A-T carriers and BMI > 24 kg/m^2^ (adjusted OR = 1.08, 95% CI = 0.75–1.54, *P* < 0.001) increased dyslipidemia risk. When gene-gene and gene-environment interactions were analyzed, we could find that the A-C-A-T-A-A-G-G-A-T-A-T and G-T-C-A-A-A-G-G-A-T-A-T carriers decreased dyslipidemia risk (adjusted OR = 0.88, 95% CI = 0.62–1.02, *P* < 0.001), whereas the G-T-C-A-A-A-G-G-A-T-A-T carriers and BMI > 24 kg/m^2^ (adjusted OR = 1.13, 95% CI = 0.85–1.49, *P* < 0.001) increased dyslipidemia risk.

**Table 5:** GMDR analysis revealed different interactions among SNPs, haplotype, gene and environment.

Locus no.	Best combination	Training Bal.Acc	Testing Bal.Acc	Cross-validation consistency	*P*	^*^ *P*
SNP-snp interactions						
2	rs2965101 rs8100239	0.7283	0.7184	10/10	<0.001	<0.001
3	rs2965101 rs157580 rs439401	0.7518	0.7494	10/10	<0.001	<0.001
SNP-environment interactions						
2	rs2965101 BMI > 24 kg/m^2^	0.7584	0.7023	9/10	<0.001	<0.001
3	rs2965101 rs8100239 BMI > 24 kg/m^2^	0.7447	0.7011	10/10	<0.001	<0.001
Haplotype-haplotype interactions						
2	P1 T4	0.6045	0.5954	10/10	0.0023	0.0018
3	P4 T1 T4	0.6482	0.6125	10/10	<0.001	<0.001
Haplotype-environment interactions						
2	T1 BMI > 24 kg/m^2^	0.6440	0.6510	9/10	<0.001	<0.001
3	T1 T4 BMI > 24 kg/m^2^	0.6807	0.6674	10/10	<0.001	<0.001
Gene-gene interactions						
2	H4 H8	0.6848	0.6544	8/10	0.082	0.065
3	H2 H4 H8	0.7324	0.6968	10/10	0.0455	0.0312
Gene-environment interactions						
2	H8 BMI > 24 kg/m^2^	0.6394	0.6220	8/10	<0.001	<0.001
3	H8 Age > 75 BMI > 24 kg/m^2^	0.7372	0.7044	9/10	<0.001	<0.001

*P* – adjusting for height, weight. *Indicates 1000 permutation tests. The haplotype is combined with *BCL3* rs2965101-rs4803748-rs2965169-rs8100239, *PVRL2* rs10402271-rs3810143-rs519113-rs6859-rs283810 and *TOMM40* rs157580-rs2070650-rs439401.

**Table 6:** Analysis for different types of interaction by using logistic regression.

Variable 1	Variable 2	OR (95% CI)	*P*-value
SNP-snp interactions			
rs157580	rs439401		
CC	TT	1	—
CC	CC+CT	0.89(0.76–1.12)	0.0323
CT+TT	TT	0.79(0.64–1.16)	0.0182
CT+TT	CC+CT	0.54(0.32–0.93)	1.7E-004
SNP-environment interactions			
rs2965101	BMI > 24 kg/m^2^		
AA	No	1	—
AA	Yes	1.14(0.98–1.37)	0.653
AC+CC	No	0.83(0.74–1.18)	0.054
AC+CC	Yes	1.08(0.84–1.44)	0.0015
Haplotype-haplotype interactions			
P1	T1		
No-carriers	No-carriers	1	—
Carriers	No-carriers	2.37(1.78–3.45)	3E-004
No-carriers	Carriers	1.64(1.03–2.88)	0.0278
Carriers	Carriers	5.47(3.64–7.73)	4.3E-005
Haplotype-environment interactions			
T4	BMI > 24 kg/m^2^		
No-carriers	No	1	—
No-carriers	Yes	1.13(0.92–1.26)	0.271
Carriers	No	1.77(1.54–2.23)	0.451
Carriers	Yes	1.08(0.75–1.54)	3.4E-005
Gene-gene interactions			
H4	H8		
No-carriers	No-carriers	1	—
Carriers	No-carriers	1.34(0.94–2.27)	1.6E-004
No-carriers	Carriers	0.76(0.55–0.97)	0.0022
Carriers	Carriers	0.88(0.62–1.02)	2.4E-005
Gene-environment interactions			
H8	BMI > 24 kg/m^2^		
No-carriers	No	1	—
No-carriers	Yes	1.22(0.98–1.21)	0.0012
Carriers	No	0.92(0.84–1.05)	0.433
Carriers	Yes	1.13(0.85–1.49)	2.7E-005

*P* – adjusting for height, weight. The haplotype is combined with *BCL3* rs2965101-rs4803748-rs2965169-rs8100239, *PVRL2* rs10402271-rs3810143-rs519113-rs6859-rs283810 and *TOMM40* rs157580-rs2070650-rs439401.

## Discussion

The main findings in the current study included: (1) it elucidated the frequencies of single nucleotide mutation, haplotype and the G × G inter-locus interaction among *BCL3, PVRL2* and *TOMM40* genes in the Maonan ethnic group, which may be proposed as an potential supplement to the 1000 Genomes database; (2) it gave some new messages about single nucleotide mutation, haplotype, G × G and G × E interaction evidence to prove there are possible interaction between the *BCL3*, *PVRL2* and *TOMM40* genes and serum lipid concentrations; (3) it detected that there were some different effects based on SNP-SNP, SNP-environment, haplotype-haplotype, haplotype-environment, gene-gene, gene-environment interaction; and (4) it found different interactions contributed to the dyslipidemia risk.

In November 2013, with an update in 2014, the American College of Cardiology (ACC) and American Heart Association (AHA) released clinical practice guidelines for the treatment of blood cholesterol to reduce cardiovascular risk^7,16^. Dyslipidemia, it was also a major modifiable risk factor for cardiovascular disease accounting for an estimated 4 million deaths per year worldwide^17,18^. According to previous study that serum lipid levels and the prevalence of dyslipidemia are determined by multiple environmental factors such as poor diet^19^, unhealthy lifestyle^20^, physical inactivity^21,22^, genetic factors^23^ and their interactions^24^.

The present study identified association of the *BCL3, PVRL2* and *TOMM40* mutations with serum lipid levels. Besides, there were significant differences in the genotypic and allelic frequencies of the 12 SNPs between the two groups. These results suggest that the prevalence of the dyslipidemia may results from genetic factors. When the relationship between SNPs and risk of dyslipidemia was analyzed, we found that the rs157580, rs439401 and rs2965101 SNPs can decrease the risk. However, SNP-environment interaction showed that the subjects with rs2965101 AC/CC and BMI > 24 kg/m^2^ lead to an increased effect on risk of dyslipidemia. Similar consequences were also found in haplotype-haplotype, haplotype-environment, gene-gene and gene-environment interactions. Maybe a reasonable explanation was that in conjunction with lifestyle and environmental factors, a genetic factor has been revealed to contribute to the development of this metabolic disorder^25,26^. Maonan people like to pickle sour meat, snails and vegetables. A typical food, Minglun Sliced Pig is a well-known dish of the Maonan ethnic group. Most of the Maonan people like to eat food which is cooked half ripe, as they believe that some kinds of vegetables and meat, especially chickens, will lose their delicious flavor if they are boiled to be too much ripe. In addition, they also like to eat beef, pork and/or animal offals in a hot pot which contain abundant saturated fatty acid^27^. Long-term high saturated fat diet is an important risk factor for obesity, dyslipidemia, atherosclerosis, and hypertension^28^. The major dietary saturated long-chain fatty acids such as myristic acid (14:0) and palmitic acid (16:0) have been associated with deleterious effects on blood lipid metabolism, especially due to their influence on plasma TC and TG levels^29^.

Unhealthy lifestyle factors such as excessive alcohol consumption and cigarette smoking have been associated with dyslipidemia^28^. In the present study, we showed that the percent of cigarette smoking and alcohol consumption were higher in dyslipidemia than in normal groups. Most of the local adult men of the Maonan people liked to drink. They even had the custom that it would be considered to be impolite to treat their guests without wines. Some families made wines themselves using grain sorghums and corns. Several previous researches have shown that alcohol may have a beneficial effect on coronary heart disease that could be mediated by elevation of HDL-C^30,31^. However, the beneficial increase in HDL-C was offset by increasing in cigarette smoking, because smoking not only increasing TC, TG, LDL-C, but also decreasing HDL-C^32–34^. That would be accounted for the current serum lipid results between the two groups. Recently, GWASes have identified numerous variants associated with lifestyle behaviors and health outcomes. However, what is sometimes overlooked is the possibility that genetic variants identified in GWAS of disease might reflect the effect of modifiable risk factors as well as direct genetic effects. We discussed this possibility with illustrative examples from tobacco and alcohol research, in which genetic variants that predict behavioral phenotypes have been seen in GWAS of diseases known to be causally related to these behaviors. This consideration has implications for the interpretation of GWAS findings^35^.

There are several limitations in our study. Firstly, the size of our study population is not big enough, which might not have the confidence to detect the interaction across the inter-locus. Next, the number of participants available for minor allele frequency (MAF) of some mutations was a little low to calculate a strong power as compared with many previous GWAS and replication studies. In addition, a lot of unmeasured environmental and genetic factors including dietary patterns, physical activities, energy intakes and so on needed to be considered. Furthermore, the relevance of this finding has to be defined in further high caliber of studies including incorporating the genetic information of *BCL3, PVRL2* and *TOMM40* gene single nucleotide mutation, haplotypes, G × G and G × E interactions *in vivo* and *in vitro* functional studies to confirm the impact of a variant on a molecular level including transcription and expression.

In conclusion, there were potential interaction between the *BCL3, PVRL2* and *TOMM40* genes, environment and serum lipid concentrations in Maonan ethnic group. And, the association analysis based on haplotype clusters and G × G interactions probably increased power over single-locus tests for the risk of dyslipidemia. When we used GMDR to analyze, different ways of interaction between gene and environment exhibited different synergy or redundancy effect on morbidity. Besides genetic factors, environment exposures would be an important point cannot be ignored.

## Materials and Methods

### Mutation selection

We selected 12 SNPs in the *BCL3, PVRL2* and *TOMM40* with the following steps: (1) *BCL3* gene clusters, which were selected from previous GWAS associated with lipid-metabolism. *PVRL2/TOMM40* gene clusters are found to be closed to *BCL3* gene and associated with serum lipid level. (2) Tagging SNPs, which were established by Haploview (Broad Institute of MIT and Harvard, USA, version 4.2) and functional SNPs predicted to lead to serum lipid changes from current version of online resource (1000 Genome Project Database). (3) SNPs information was obtained from NCBI dbSNP Build 132 (http://www.ncbi.nlm.nih.gov/SNP/); (4) SNPs were restricted to minor allele frequency (MAF) > 1%; and (5) SNPs might be associated with the plasma lipid levels or cardiovascular disease in recent studies (6) *BCL3* rs2965101-rs4803748-rs2965169-rs8100239, *PVRL2* rs10402271-rs3810143-rs519113-rs6859-rs283810 and *TOMM40* rs157580-rs2070650-rs439401, which were selected by the block-based approach. This strategy is enable by the correlations between tagging SNPs as manifested as LD (*r*^*2*^ > 0.8). Although classic is not goal of tagging SNP selection, innovative tagging SNPs selection bias is inevitable.

### Ethical approval

The study was carried out following the rules of the Declaration of Helsinki of 1975 (http://www.wma.net/en/30publications/10policies/b3/), revised in 2008. All participants from contributing populations gave written informed consent to participate in epidemiologic investigation and genetic analysis. All study protocols in this motif have approval from the Ethics Committee of the First Affiliated Hospital, Guangxi Medical University (No: Lunshen-2011-KY-Guoji-001; Mar. 7, 2011).

### Subjects

Two groups of study population including 1962 participants of Maonan (744 males, 37.92% and 1218 females, 62.08%) were randomly selected from our previous stratified randomized samples^36^. All participants were resided in the Huanjiang Maonan Autonomous County in the Northwestern of Guangxi Zhuang Autonomous Region, which is located in Southwestern China. The participants’ age ranged from 18 to 80 years with a mean age of 56.37 ± 10.78 years in normal and 57.22 ± 11.12 years in dyslipidemia; respectively. The gender ratio and age distribution were matched between the two groups. All participants were essentially healthy with no history of coronary artery disease, stroke, diabetes, hyper- or hypo-thyroids, and chronic renal disease. They were free from medications known to affect lipid profiles.

### Epidemiological survey

The epidemiological survey was carried out using internationally standardized method, following a common protocol^37^. Information on demographics, socioeconomic status, and lifestyle factors were collected with standardized questionnaires. Cigarette smoking status was categorized into groups of cigarettes per day: ≤20 and >20^38^. Alcohol consumption was categorized into groups of grams of alcohol per day: ≤25 and >25^39^. Several parameters such as blood pressure, height, weight and WC were measured, while BMI (kg/m^2^) was calculated.

### Biochemical measurements

Venous blood samples were obtained from all subjects after at least 12 h of fasting. The levels of serum TC, TG, HDL-C, and LDL-C in samples were determined by enzymatic methods with commercially available kits, Tcho-1, TG-LH (RANDOX Laboratories Ltd., Ardmore, Diamond Road, Crumlin Co., Antrim, UK, BT29 4QY), Cholestest N HDL, and Cholestest LDL (Daiichi Pure Chemicals Co., Ltd., Tokyo, Japan), respectively. Serum ApoA1 and ApoB levels were detected by the immunoturbidimetric immunoassay (RANDOX Laboratories Ltd.). All determinations were performed with an autoanalyzer (Type 7170A; Hitachi Ltd., Tokyo, Japan) in the Clinical Science Experiment Center of the First Affiliated Hospital, Guangxi Medical University^40^.

### Diagnostic criteria

The normal values of serum TC, TG, HDL-C, LDL-C, ApoA1, ApoB levels and the ApoA1/ApoB ratio in our Clinical Science Experiment Center were 3.10–5.17, 0.56–1.70, 1.16–1.42, 2.70–3.10 mmol/L, 1.20–1.60, 0.80–1.05 g/L and 1.00–2.50, respectively. Dyslipidemia was defined according to World Health Organization criteria: TG ≥ 1.7 mmol/L and HDL-C < 0.9 mmol/L for men or <1.0 mmol/L for women^41–43^. Hypertension was diagnosed according to the 1999 and 2003 criteria of the World Health Organization-International Society of Hypertension Guidelines for the management of hypertension. The diagnostic criteria of overweight and obesity were according to the Cooperative Meta-analysis Group of China Obesity Task Force. Normal weight, overweight and obesity were defined as a BMI < 24, 24–28 and >28 kg/m^2^, respectively.

### Genotyping

Genomic DNA was extracted from leucocytes of venous blood using the phenol-chloroform method. Genotyping of 12 mutations was performed by PCR and Sanger sequencing. The characteristics of each mutation and the details of each primer pair, annealing temperature, length of the PCR products are summarized in Supplemental Tables. The PCR products of the samples were sequenced with a sequencer ABI Prism 3100 Genetic Analyzer (Applied Biosystems, International Equipment Trading Ltd., Vernon Hills, IL, USA) in Shanghai Sangon Biological Engineering Technology & Services Co. Ltd., Shanghai China.

### Statistical analyses

The statistical analysis was performed with the statistical software SPSS 22.0 (SPSS Inc., Chicago, IL, USA). Quantitative variables were presented as the mean ± SD for those, that are normally distributed, whereas the medians and interquartile ranges for TG, which is not normally distributed. General characteristics between the two groups were compared by the *ANCOVA*. The distributions of the genotype, allele, haplotype and G × G interaction between the two groups were analyzed by the chi-squared test; the HWE, Pair-wise LD, frequencies of haplotype and G × G interaction comprising the mutations were calculated using Haploview (version 4.2; Broad Institute of MIT and Harvard). The pattern of pair-wise LD between the selected mutations was measured by *D′* using the Haploview software. The association of the genotypes, haplotypes and G × G interactions with lipid phenotypic variations was tested by the *Univariant*. Any variants associated with the lipid phenotypic variations at a value of *P* < 0.004 (corresponding to *P* < 0.05 after adjusting for 12 independent tests by the Bonferroni correction) were considered statistically significant. Unconditional logistic regression was used to assess the association of the genotypes (common homozygote genotype = 1, heterozygote genotype = 2, rare homozygote genotype = 3), alleles (the minor allele non-carrier = 1, the minor allele carrier = 2), haplotypes (the haplotype non-carrier = 1, the haplotype carrier = 2) and G × G interactions (the G × G interaction non-carrier = 1, the G × G interaction carrier = 2) with lipid phenotypic variations. The model of age, gender, BMI, WC, SBP, DBP, pulse pressure, cigarette smoking, alcohol consumption and fasting plasma glucose level were adjusted for the statistical analysis. Generalized multifactor dimensionality reduction (GMDR)^44^ was used to screen the best interaction combination among genes, SNPs and *environmental* exposures. The cross-validation consistency score was a measure of the degree of consistency with which the selected interaction was identified as the best model among all possibilities considered. The testing balanced accuracy was a measure of the degree to which the interaction accurately predicts case-control status with scores between 0.50 (indicating that the model predicts no better than chance) and 1.00 (indicating perfect prediction). Finally, a sign test or a permutation test (providing empirical *P*-values) for prediction accuracy can be used to measure the significance of an identified model.

## Electronic supplementary material

Supplementary Information
