# Supplementary material for: BCL3-PVRL2-TOMM40 SNPs, gene-gene and gene-environment interactions on dyslipidemia
Source: Sci Rep. 2018 Apr 18;8:6189. doi: 10.1038/s41598-018-24432-w (PMC5906470; doi:10.1038/s41598-018-24432-w)
Supplement: Supplementary file 1 — Supplementary Information [file 41598_2018_24432_MOESM1_ESM.doc]

***BCL3-PVRL2-TOMM40* SNPs, gene-gene and gene-environment interactions on dyslipidemia**

Liu Miao^1^, Rui-Xing Yin^1^, Shang-Ling Pan^2^, Shuo Yang^1^, De-Zhai Yang^3^ & Wei-Xiong Lin^3^

^1^ Department of Cardiology, Institute of Cardiovascular Diseases, The First Affiliated Hospital, Guangxi Medical University, Nanning 530021, Guangxi, People’s Republic of China. ^2^ Department of Pathophysiology, School of Premedical Science, Guangxi Medical University, Nanning 530021, Guangxi, People’s Republic of China. ^3^ Department of Molecular Genetics, Medical Scientific Research Center, Guangxi Medical University, Nanning 530021, Guangxi, People’s Republic of China.

**Running title:** *BCL3-PVRL2-TOMM40* variants and dyslipidemia

Correspondence and requests for materials should be addressed to R.-X.Y. (email: yinruixing@163.com)

dr.miaoliu@qq.com

yinruixing@163.com

shanglingpan@hotmail.com

yangshuo1112@outlook.com

ydz969@126.com

lin78018@163.com

**Supplemental Table 1** Characteristics of the *BCL3, PVRL2* and *TOMM40* mutations.

| **SNV ID (rs#)** | **HGVS Name** | **Chr: Position** | **Contig** | **Contig Pos** | **SNP to Chr** | **Allele** | **MAF/Minor** | **Map**  **Methods** |
| --- | --- | --- | --- | --- | --- | --- | --- | --- |
| ***BCL3*** |  |  |  |  |  |  |  |  |
| rs2965101 | NC_000019.10: g.44734556A>G | 19:44734556 | NT_011109.17 | 17493862 | Rev | G | T=0.3934/1970 (1000 Genomes) | mapup |
| rs4803748 | XM_005259129.1: c.108+867C>T | 19:44743791 | NT_011109.17 | 17502917 | Fwd | C | C=0.4696/2352 (1000 Genomes) | mapup |
| rs2965169 | NM_005178.4: c.-892A>C | 19:44747899 | NT_011109.17 | 17507025 | Fwd | A | A=0.4655/2331 (1000 Genomes) | mapup |
| rs8100239 | XM_005259129.1: c.844+801A>T | 19:44749847 | NT_011109.17 | 17508973 | Fwd | T | T=0.3926/1966  (1000 Genomes) | mapup |
| ***PVRL2*** |  |  |  |  |  |  |  |  |
| rs10402271 | NC_000019.10: g.44825957A>C | 19: 44825957 | NT_011109.17 | 17585083 | Fwd | C | C=0.2332/1168 (1000 Genomes) | mapup |
| rs3810143 | NM_001042724.1: c.-381A>G | 19: 44846145 | NT_011109.17 | 17605271 | Fwd | G | G=0.3670/1838  (1000 Genomes) | mapup |
| rs519113 | NM_001042724.1: c.775+878C>G | 19: 44873027 | NT_011109.17 | 17632153 | Rev | G | G=0.2668/1336  (1000 Genomes) | mapup |
| rs6859 | NM_001042724.1: c.1043-3434A>G | 19:44878777 | NT_011109.17 | 17637903 | Fwd | A | A=0.3664/1835 (1000 Genomes) | mapup |
| rs283810 | NM_001042724.1: c.1197-953A>C | 19: 44884984 | NT_011109.17 | 17644110 | Rev | C | C=0.1775/889  (1000 Genomes) | mapup |
| ***TOMM40*** |  |  |  |  |  |  |  |  |
| rs157580 | NM_001128916.1: c.274+320C>T | 19:44892009 | NT_011109.17 | 17651135 | Fwd | C | C=0.3486/1746 (1000 Genomes) | mapup |
| rs2075650 | NM_001128916.1: c.275-31A>G | 19:44892362 | NT_011109.17 | 17651488 | Fwd | G | G=0.1194/598 (1000 Genomes) | mapup |
| rs439401 | NG_007084.2: g.10413T>C | 19:44911194 | NT_011109.17 | 17670320 | Fwd | C | T=0.4050/2028 (1000 Genomes) | mapup |

**Supplemental Table 2** The sequences of forward and backward primers of the *BCL3, PVRL2* and *TOMM40* mutations.

| **SNV** | **Primer sequence** | **Annealing temperature** | **PCR product** |
| --- | --- | --- | --- |
| ***BCL3*** |  |  |  |
| rs2965101 | CCAAGCATAAGAGAATCTTAAGGAC | 59°C | 468bp |
|  | GGTTCTGGAATCCCTTGTTCCT |  |  |
| rs4803748 | ACCCTCCCAAGTAGCTAGGA | 60°C | 448bp |
|  | TTGGGAGGCCAAGGTAGAAG |  |  |
| rs2965169 | CCCTCTTCCATCTCTGCCAC | 62°C | 541bp |
|  | CTCAGTGACCCGGACTCAAC |  |  |
| rs8100239 | CCTTTACTGCGGTCCAGGGC | 60°C | 401bp |
|  | CCTCCCTCCTGCCTCTCTT |  |  |
| ***PVRL2*** |  |  |  |
| rs10402271 | AGACAGACAGAGACAGAGCC | 60°C | 390bp |
|  | ACAGCCTTCCCCAGTCTAAC |  |  |
| rs3810143 | AGGGTACGAGAAACAGGCTC | 61°C | 430bp |
|  | GGATTCTAAGGTCGCCCCTG |  |  |
| rs519113 | GTCAAAACAGGGTTTGGGCA | 60°C | 644bp |
|  | ACATCGTCTCCACACCTTCC |  |  |
| rs6859 | AGACAGACAGAGACAGAGCC | 60°C | 464bp |
|  | ACAGCCTTCCCCAGTCTAAC |  |  |
| rs283810 | CGTCAGCAAGGTGTCTCCAG | 62°C | 382bp |
|  | CTCCATGCTGTGACCCACTG |  |  |
| ***TOMM40*** |  |  |  |
| rs157580 | TTTCTTCTGTCCCTCCC | 58°C | 351bp |
|  | CACATTCGAGGAGTGCCACC |  |  |
| rs2075650 | CGAGGTTCCTTGGGTATGGG | 62°C | 493bp |
|  | GGGACGATCTCTGGTGGGTA |  |  |
| rs439401 | CCTACTGGGTGCTGAATCCA | 59°C | 117bp |
|  | CTCCCCGTCTCTGAGAACTG |  |  |


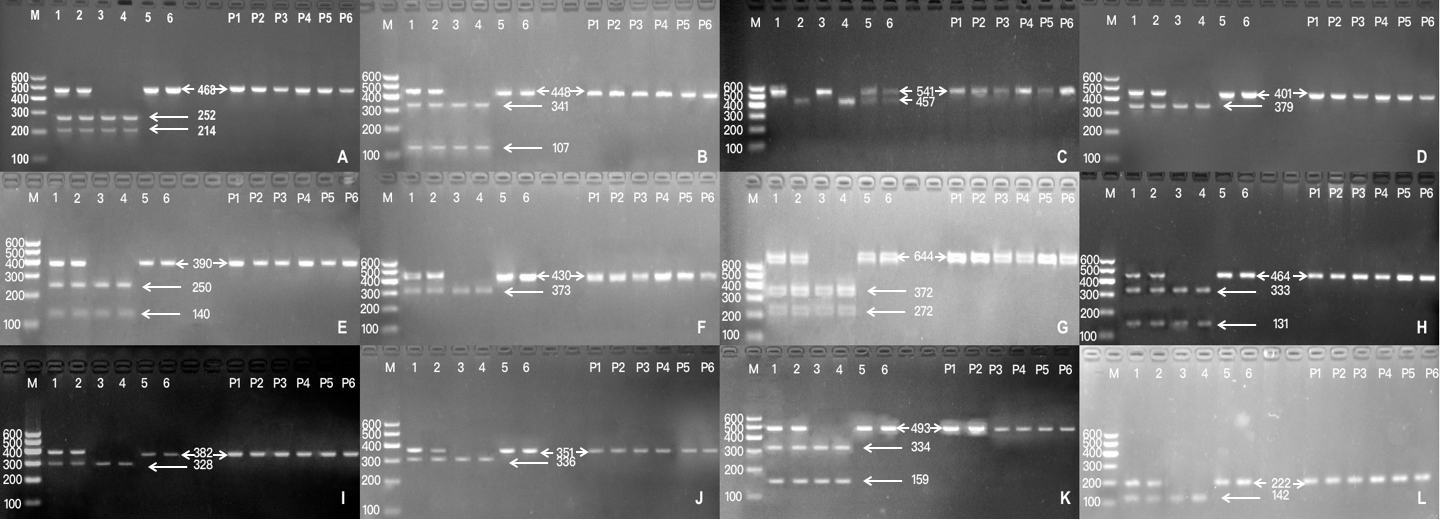


**Supplemental Figure Agarose gel electrophoresis (2%) of PCR products of the *BCL3, PVRL2* and *TOMM40* SNPs.** Lane M: DNA ladder100bp. The genotypes of 12 SNPs were as follow: (A) *BCL3* rs2965101 A > G AA (Lane 5 and 6), AG (Lane 1 and 2), GG (Lane 3 and 4), (B) *BCL32* rs4803748 C > T, CC (Lane 5 and 6), CT (Lane 1 and 2), TT (Lane 3 and 4), (C) *BCL3* rs2965169 A > C, AA (Lane 1 and 3), AC (Lane 5 and 6), CC (Lane 2 and 4),(D) *BCL3* rs8100239 A > T, AA (Lane 5 and 6), AT (Lane 1 and 2), GG (Lane 3 and 4), (E) *PVRL2* rs10402271 A > C, AA (Lane 5 and 6), AC (Lane 1 and 2), CC (Lane 3 and 4), (F) *PVRL2* rs3810143 A > G, AA (Lane 5 and 6), AG (Lane 1 and 2), GG (Lane 3 and 4), (G) *PVRL2* rs519113 C > G, CC (Lane 5 and 6), CG (Lane 1 and 2), GG (Lane 3 and 4), (H) *PVRL2* rs6859 A > G, AA (Lane 5 and 6), AG (Lane 1 and 2), GG (Lane 3 and 4), (I) *PVRL2* rs283810 A > C, AA (Lane 5 and 6), AC (Lane 1 and 2), CC (Lane 3 and 4), (J) *TOMM40* rs157580 C > T, CC (Lane 5 and 6), CT (Lane 1 and 2), TT (Lane 3 and 4), (K) *TOMM40* rs2075650 A > G, AA (Lane 5 and 6), AG (Lane 1 and 2), GG (Lane 3 and 4), (L) *TOMM40* rs439401 C > T, CC (Lane 5 and 6), CT (Lane 1 and 2), TT (Lane 3 and 4), DNA ladder cannot be seen when it less 100bp. Lane P1-P6: PCR amplicon of SNPs were 468-, 448-, 541-, 401-, 390-, 430-, 644-, 464-, 382-, 351-, 493- and 222-bp nucleotide sequences; respectively.
